# Rationale and design of the CAROLINA® - cognition substudy: a randomised controlled trial on cognitive outcomes of linagliptin versus glimepiride in patients with type 2 diabetes mellitus

**DOI:** 10.1186/s12883-018-1014-7

**Published:** 2018-01-15

**Authors:** Geert Jan Biessels, Jolien Janssen, Esther van den Berg, Bernard Zinman, Mark A. Espeland, Michaela Mattheus, Odd Erik Johansen, on behalf of the CAROLINA® investigators

**Affiliations:** 1 Department of Neurology, G03.232 Brain Center Rudolf Magnus, University Medical Center, PO Box 85500, 3508 GA Utrecht, the Netherlands; 2 Julius Center for Health Sciences and Primary Care, University Medical Center Utrecht, Utrecht, the Netherlands; 3 Department of Neurology, Erasmus MC - University Medical Center, Rotterdam, the Netherlands; 4 Lunenfeld-Tanenbaum Research Institute, Mount Sinai Hospital, Toronto, Canada, Division of Endocrinology, University of Toronto, Toronto, Canada; 5 Department of Biostatistical Sciences, Wake Forest School of Medicine, Winston-Salem, NC USA; 6 Global biometrics and datamanagement, Boehringer Ingelheim, Ingelheim, Germany; 7 Clinical development, Therapeutic Area Metabolism, Boehringer Ingelheim, Asker, Norway

**Keywords:** Type 2 diabetes mellitus, Cognition, Dipeptidyl peptidase-IV inhibitor, Oral glucose-lowering agent, Dementia

## Abstract

**Background:**

Type 2 diabetes mellitus is associated with cognitive dysfunction and an increased risk of dementia. Linagliptin is a glucose-lowering agent of the dipeptidyl peptidase-IV (DPP-IV) inhibitor class that is of particular interest for the prevention of accelerated cognitive decline, because it may potentially benefit the brain through pleiotropic effects, beyond glucose lowering. This paper presents the design of a study that aims to establish if linagliptin is superior to the sulfonylurea glimepiride in the prevention of accelerated cognitive decline in patients with type 2 diabetes mellitus.

**Methods:**

The cognition substudy is an integral part of the ongoing event-driven, randomised, double blind CARdiOvascular safety of LINAgliptin (CAROLINA®) trial, which evaluates the effect of treatment with linagliptin versus glimepiride on cardiovascular outcomes. CAROLINA® includes patients with type 2 diabetes mellitus with sub-optimal glycaemic control at elevated cardiovascular risk. The substudy will evaluate patients randomised and treated who have a baseline Mini Mental State Examination (MMSE) score ≥ 24, documented years of formal education with at least one valid cognitive assessment at baseline and during follow-up. The primary cognitive outcome is the occurrence of accelerated cognitive decline at the end of follow-up. The two treatment groups will be compared by using a logistic regression. Accelerated cognitive decline is defined as a rate of cognitive decline that falls at or below the 16th percentile of decline for the whole cohort on either the MMSE or a combined score of the trail making and verbal fluency test. Potential confounders are taken into account at an individual patient level, using a regression based index.

**Discussion:**

Between December 2010 and December 2012, 6042 patients were randomised and treated with either linagliptin (5 mg) or glimepiride (1-4 mg) once daily in CAROLINA®. Cognitive tests were conducted in nearly 4500 participants at baseline and are scheduled for two subsequent assessments, after 160 weeks of follow-up and end of follow-up. This substudy of the ongoing CAROLINA® trial will establish if linagliptin is superior to glimepiride in the prevention of accelerated cognitive decline in patients with type 2 diabetes mellitus. Final results are expected in 2019.

**Trial registration:**

ClinicalTrials.gov Identifier: NCT 01243424.

**Electronic supplementary material:**

The online version of this article (10.1186/s12883-018-1014-7) contains supplementary material, which is available to authorized users.

## Background

Type 2 diabetes mellitus (T2DM) is a rising public health concern with over 400 million cases worldwide in 2015 and an estimated number of over 600 million cases by 2040 [1]. Prevention of long-term complications is a major focus of diabetes treatment. In this respect, cognitive dysfunction and dementia are diabetes-associated complications that receive increasing attention [2, 3]. It is well recognised that the risk of dementia is increased in people with T2DM [4]. A recent meta-analysis evaluated 20 studies reporting on the risk of any type of dementia, 20 on Alzheimer’s disease and 13 on vascular dementia (VaD), including a total of 1,148,041 participants, of whom 89,708 had diabetes. The pooled relative risk (95% CI) for dementia in people with diabetes was 1.73 (1.65–1.82), for Alzheimer’s disease 1.56 (1.41–1.73) and for VaD 2.27 (1.94–2.66) [5] as compared to people without. In addition, diabetes is associated with more subtle cognitive changes, that are referred to as diabetes-associated cognitive decrements [2, 3].

Accelerated cognitive decline is a cause for concern in patients with T2DM, yet no preventive treatment has been established. Lifestyle, vascular, and diabetes-specific risk factors present many promising targets for prevention and treatment [2, 6, 7]. These include management of glycaemic control and avoidance of severe hypoglycaemic events [8]. Previous observational studies that examined the effect of glucose-lowering treatments (including metformin, sulfonylureas, thiazolidinedione, insulin or a combination of these) on the risk of cognitive decline have not demonstrated consistent findings [2]. Because observational studies have a substantial risk of bias, randomised controlled trials (RCTs) are needed; unfortunately few have been performed. A recent meta-analysis summarised the results of five well conducted RCTs on the effect of intensive versus standard glycaemic control on cognitive decline in patients with T2DM, involving over 24,000 participants [9]. This pooled analysis showed that intensive glycaemic control was not associated with a slower rate of cognitive decline, compared with standard glycaemic control, although there was some heterogeneity among studies [9]. These previous RCTs have in common that they used mean cognitive performance as their primary outcome, which may include many participants with little or no cognitive decline. Although duration of follow-up of the studies ranged from 3 to 6 years [9], the actual average decline in mean cognitive performance was limited [10–13]. Over the past years it has become clear, also from observational studies, that the average decline in cognition over time associated with diabetes [2] is relatively slow, limiting the sensitivity of follow up studies to detect meaningful differences. Importantly however, among patients with T2DM there is heterogeneity in the rate of cognitive decline, where some have accelerated decline which in some cases progress to dementia. For example, in a large cohort of patients with T2DM over the age of 60 years, annual incidence of dementia of 2.6% was reported [14]. It might therefore be more appropriate - and clinically meaningful with regards to establishing interventions - to focus on occurrence of accelerated cognitive decline in individual patients. Such an approach is chosen in the CAROLINA®-cognition substudy. Interestingly, the ORIGIN study (which studied effects on outcomes of intensive glucose lowering with insulin glargine) did a post-hoc analysis using this approach in the ORIGIN MIND substudy and observed a modest, albeit statistically non-significant, benefit of intensive glycaemic control [10] versus standard care.

Dipeptidyl peptidase-IV (DPP-IV) inhibitors improve glycaemic control by inhibiting the enzyme DPP-IV thereby enhancing the incretin effects, i.e., increasing the availability of active glucagon-like peptide (GLP)-1 and glucose-dependent insulinotropic polypeptide (GIP), which are secreted from the intestine after a meal. In the presence of hyperglycaemia, these hormones promote glucose-dependent insulin secretion and reduce glucagon secretion [15]. Beyond their effects on DPP-IV activity and glucose, several preclinical studies suggest anti-inflammatory, anti-atherosclerotic and neuroprotective effects that might be relevant in the context of preventing accelerated cognitive decline [15–19]. Experimental studies also show promising results of incretin-based therapies in models of Alzheimer’s disease and stroke [17]. These potential pleiotropic modes of action make DPP-IV inhibitors attractive candidate drugs to prevent accelerated cognitive decline in T2DM. Recently, an observational study found that increased plasma DPP-IV activity was associated with a high risk of mild cognitive impairment in elderly patients with T2DM [20], providing further support to test a strategy of modulating DPP-IV activity in T2DM to prevent cognitive impairment. The international, randomised, double blinded CARdiOvascular safety of LINAgliptin (CAROLINA®) trial is designed to provide a long-term evaluation of treatment durability and cardiovascular safety of treatment with the DPP-IV inhibitor linagliptin compared to the currently widely used sulfonylurea (SU) glimepiride [21, 22]. Linagliptin is a once-daily, DPP-IV inhibitor with a xanthine-based structure that is characterised by a pharmacological profile distinct from other drugs in this class [23] largely due to its non-renal route of elimination (80% hepatic versus 5% renal) [24]. The cognition substudy is an integrated part of CAROLINA®.

### Objectives

The primary objective of the CAROLINA®-cognition substudy is to investigate if the proportion of participants with accelerated cognitive decline is lower in the group randomised to treatment with linagliptin compared to the group randomised to glimepiride after 160 weeks, or at end of follow-up.

#### Secondary objectives

Unravelling the processes that underlie cognitive decline in T2DM is important to support future prevention strategies. Secondary objectives are therefore:

1. At baseline: to explore associations between characteristic features of T2DM (i.e., glycaemic and anthropometric parameters), cardiovascular risk factors (i.e., blood pressure and lipid levels) and cognitive performance
2. Longitudinal: to explore associations between baseline characteristic features of T2DM, cardiovascular risk factors – and changes in these factors over time – and cognitive decline during follow-up
3. Longitudinal: to explore the associations between baseline mood – and changes in mood over time - and cognitive decline during follow-up

## Methods

### Design and sample

The CAROLINA® trial is a randomised, active comparator, double blind study to evaluate the cardiovascular safety of linagliptin versus glimepiride in patients with T2DM at elevated cardiovascular risk. Patients were randomised between 2010 and 2012 from approximately 600 trial centres in 43 different countries. Key inclusion criteria are shown in Table 1.

**Table 1:** Key **i**nclusion criteria CAROLINA®

Insufficient glycaemic control defined as one of the criteria (A or B)	AND	Elevated risk of cardiovascular events defined as any (one or more) of the criteria (A, B, C or D)
**(A) HbA1c 6.5 - 8.5% (48–69 mmol/mol) while patient is treatment naïve or treated with:** (I) Metformin monotherapy (II) α-Glucosidase inhibitor monotherapy (e.g. acarbose, voglibose) (III) Metformin plus α-glucosidase inhibitor (e.g. acarbose, voglibose) **(B) HbA1c 6.5 - 7.5% (48–58 mmol/mol) while patient is treated with:** (I) SU monotherapy (II) Glinide monotherapy (e.g. repaglinide, nateglinide) (III) Metformin plus SU (for a maximum of 5 years) (IV) Metformin plus glinide (for a maximum of 5 years) (V) α-Glucosidase inhibitor plus SU (for a maximum of 5 years) (VI) α-Glucosidase inhibitor plus glinide (for a maximum of 5 years)		**(A) Previous vascular disease:** (I) MI (>6 weeks prior to informed consent IC) (II) Documented coronary artery disease (⩾50% luminal diameter narrowing of left main coronary artery or in at least two major coronary arteries in angiogram) (III) Percutaneous coronary intervention (>6 weeks prior to IC) (IV) Coronary artery bypass grafting (>4 years prior to IC) or with recurrent angina following surgery (V) Ischaemic or haemorrhagic stroke (>3 months prior to IC) (VI) Peripheral occlusive arterial disease **(B) Evidence of vascular-related end-organ damage:** (I) Moderately impaired renal function (as defined by MDRD formula) with eGFR 30–59 ml/min/1.73 m2 (II) Random spot urinary albumin:creatinine ratio ⩾30 μg/mg in two of three unrelated specimens in the previous 12 months. (III) Proliferative retinopathy defined as retinal neovascularization or previous retinal laser coagulation therapy **(C) Age ≥ 70 years** **(D) At least two of the following cardiovascular risk factors:** (I) T2DM duration >10 years (II) Systolic BP > 140 mmHg (or on at least 1 BP-lowering treatment) <6 months prior to IC (III) Current daily cigarette smoking (IV) LDL-cholesterol ⩾ 135 mg/dL (3.5 mmol/L) (or specific current treatment for this lipid abnormality) <6 months prior to IC

Table adapted from Marx et al. 2015 [21]. CAROLINA: CARdiovascular Outcome Trial of LINAgliptin Versus Glimepiride in Type 2 Diabetes; IC: informed consent; T2DM: type 2 diabetes mellitus; BP: blood pressure; SU: sulphonylurea; MI: myocardial infarction; MDRD: modified diet in renal disease; eGFR: estimated glomerular filtration rate

CAROLINA® is an event driven study. It is planned to run until a minimum of 631 confirmed Major Adverse Cardiovascular Events (MACE) have been accrued. MACE include cardiovascular death (including fatal stroke and fatal myocardial infarction (MI)), non-fatal MI (excluding silent MI) and non-fatal stroke. The estimated study duration is about 432 weeks. For more detailed information about the CAROLINA® main study see the Boehringer Ingelheim trial protocol (1218.74, Clintrial.gov id NCT01243424) and the previously published paper on the design and baseline characteristics [21].

#### CAROLINA cognition substudy

**Fig. 1:**
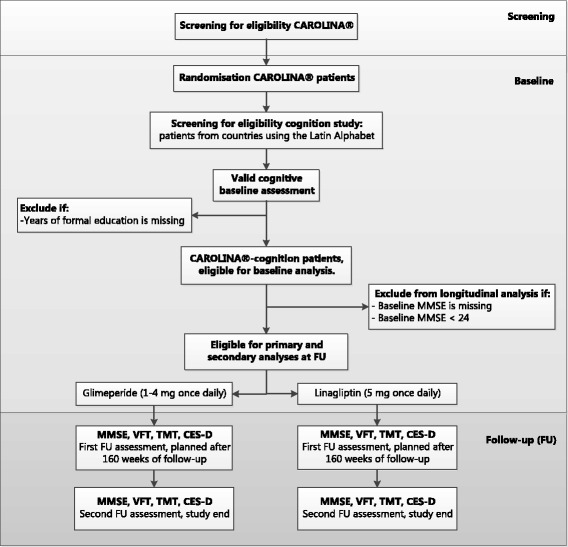
overview design CAROLINA®-cognition substudy. Abbreviations: FU: follow-up, A&E score: Attention and Executive functioning score, MMSE: Mini Mental State Examination, VFT: Verbal Fluency Test, TMT: Trail Making Test, CES-D: Centre for Epidemiologic Studies Depression Scale

Cognitive tests are conducted at baseline, after 160 weeks and at planned end of follow-up (or at permanent treatment-discontinuation). To be eligible for cognitive testing in this substudy, participants need to live in a country that have a native language built on the Latin alphabet, due to psychometric test-battery validation. Participants are included in the analysis data-set of the CAROLINA®-cognition substudy of baseline data if they are randomised and treated with at least one dose of study drug and have at least one valid cognitive assessment at baseline and documented years of formal education. For the analyses of follow-up data in addition at least one valid cognitive assessment during follow-up and baseline Mini Mental State Examination (MMSE) score ≥ 24 is required as illustrated in Fig. 1. An overview of the CAROLINA cognition substudy according to the "SPIRIT checklist" is provided in Additional file 1.

### Cognitive assessment and psychometric tests

This cognitive assessment included a cognitive paper based test battery that is brief and easy to administer in a standardised way. The tests are sensitive to relatively mild cognitive changes in T2DM, well standardised and validated, and available in multiple languages (using the modern Latin alphabet). The specific tests selected were:

1. Mini Mental State Examination (MMSE). The MMSE is a screening instrument that was developed to determine whether older adults have cognitive impairments [25]. It consists of a range of items assessing orientation, memory for words, drawing, backward counting and semantic knowledge, with a maximum score of 30. The MMSE takes approximately five minutes to administer and participating centres use country-specific validated questionnaires of the MMSE. A cut-off of <24 is widely used, and has been accepted, as indicating the presence of cognitive impairment [26]. A limitation of the MMSE is that it is insensitive to cognitive decrements in domains affected by vascular-related cognitive impairment, in particular attention, executive functioning and information processing speed [27]. Therefore two additional tests that tap into these domains were included - the Trail Making Test (TMT) and the verbal fluency test (VFT). Although the TMT and the VFT measure different cognitive processes, there is a clear consensus in cognitive theory and clinical practice that both tests assess important aspects of speed, attention and executive functioning [28].
2. TMT. The TMT is a test of scanning, visuomotor tracking, divided attention and cognitive flexibility [29]. The test requires a subject to ‘connect-the-dots’ of 25 consecutive targets on a sheet of paper. Two versions are available: A, in which the targets are all numbers (1,2,3, etc.), and B, in which the subject alternates between numbers and letters (1, A, 2, B, etc.). The goal is to finish the test as quickly as possible, and the time taken to complete the test is recorded. The maximum score (i.e. 300 s) is assigned to patients who are unable to complete the test within five minutes. The TMT is highly sensitive to the presence of cognitive impairment [30]. The TMT B is sensitive to T2DM-associated cognitive decrements, and in older individuals test performance clearly decreases over time [31–33]. The English versions of the TMT test instructions were translated into the local languages. Potential effects of translation of the test instructions on test difficulty, although unlikely, cannot be ruled out a priori and therefore will be tested (see sensitivity analyses, Table 2).
3. VFT. The VFT requires a subject to generate as many words as possible in 60 s. The category version (semantic fluency) requires generation of words from a certain category (e.g. animals), the letter version (phonemic fluency) requires generation of words starting with a specific letter. The tests are sensitive to the effects of ageing and performance is clearly affected in T2DM [27, 32, 33]. It is viewed as a sensitive indicator of (even mild) cognitive dysfunction. In CAROLINA, the category animals and the letters F, A and S are used for all languages. The number of words/animals after 15 s and after 60 s are recorded. The test takes approximately five minutes to complete. The English versions of the test instructions were translated into the local languages. Because of word-frequency differences between different Latin-based languages the letters FAS will not yield identical performance in different languages. However, FAS-equivalent letter combinations were available in a minority of languages only. Therefore, we chose to calculate a language-specific correction score (see analysis).

**Table 2:** Sensitivity analyses for the primary outcome

*Reason sensitivity analysis*	How is the sensitivity analysis performed?
Check the influence of inappropriate inclusion, potentially confounding co-morbid conditions and trial medication use	Participants will be excluded from the analysis if: • major inclusion or exclusion criteria are violated • incorrect trial medication is taken • major neurological or psychiatric disease was present at baseline
Check the influence of classifying participants who did not understand the instructions at follow-up as having accelerated cognitive decline	The last observation carried forward method will be used for patients with missing MMSE and A&E RBI-scores at follow-up if the reason for missing is the inability of the patient to understand the instructions (instead of classifying them as having accelerated cognitive decline)
Check for bias by differential lost to follow-up (worst case scenario)	All patients with missing MMSE and A&E RBI-scores at follow-up will be considered to have accelerated cognitive decline
Investigate the impact of further baseline variables on the RBI score result, Check for confounding by depression symptoms	Age, gender, years of formal education, race, ethnicity and language and CES-D (score < 16, ≥16) are included as covariates in the logistic regression analysis

For the purpose of assessing effects on Attention and Executive functioning, the TMT and the VFT are combined to one composite score for Attention and Executive functioning (the A&E score). The A&E score is calculated as follows:

1. The VFT scores for the letters F, A and S in 60 s are averaged to one VFT letter fluency score.
2. The VFT is corrected for language influences by calculating least square (LS) means in an analysis of covariance (ANCOVA) model including age, gender, years of formal education, race and language as independent variables. The LS means for language are derived and then compared to one reference language (English), i.e. correction factors are calculated for each language separately (LSmean language/ LSmean English). Correction factors will be calculated for the three letters F, A and S taken together, and for the category fluency (i.e. animals) separately. The VFT scores of each participant are then corrected by multiplying the score with the corresponding correction factor. After correction, the scores are converted into z-scores. Z-scores are used to standardise raw test scores and make them directly comparable, z-scores are calculated as follows: (individual raw test score – mean baseline test score study population)/ baseline standard deviation.
3. The corrected VFT letter fluency and the VFT category fluency z-scores (both after 60 s) are averaged to one VFT overall score, where the letter fluency and the category fluency each account for 50%.
4. The TMT ratio is calculated, providing an index for executive functioning: (TMT B –TMTA) / TMT A.
5. The TMT ratio and VFT overall score are converted into z-scores.
6. The mean of the TMT ratio and VFT overall z-scores is used to generate one composite score for attention and executive functioning. In secondary analysis the TMT and VFT will be analysed separately to control for potential test-specific effects.

As depression is a confounder to cognitive performance, participants also complete a depression questionnaire. In the CAROLINA® cognition substudy, we use the Centre for Epidemiologic Studies Depression Scale (CES-D), a widely used and validated 20-item questionnaire on depressive symptoms over the past week [34]. A score of ≥16 is indicative of a depression [35]. Whenever available in a county, the validated version of the CES-D was used. For languages were no validated version was available, a back translation was created and verified.

As both too high or too low blood glucose values can affect cognitive performance, self-monitoring of blood glucose (SMBG) values levels are to be measured (finger prick) prior to each cognitive assessment. Whenever the SMBGis not within 4 – 13 mmol/L the cognitive assessment is postponed. If values >3 or <18 mmol/L the finger prick could be repeated after at least one hour provided that the SMBGis within the 4–13 mmol/L range. In case glucose values ≤3 or ≥18 mmol/L glycemic management should be reviewed and the assessment postponed 1–7 days.

To optimise the quality of the cognitive outcomes, face-to-face meetings including training for examiners were organised in conjunction with the study start-up meetings. In addition, written step-by-step instructions for the (preparation of the) test assessment were provided. All tests were administered by the investigator or designated site-personnel who were all fluent in the language of test administration. The language in which the tests are performed is captured in the CRF. It is also recorded whether this language is the native language of the patient. If the tests are not performed in patient’s native language the VFT scores are considered to be invalid and are set to missing.

The investigator or designated site-personnel can add a comment to the test score if they doubt the validity of the test. All those comments are independently reviewed by two members of the analysis team and categorised into whether those have an impact on the test score results (“valid” or “not valid” test score results). Discrepancies are resolved by means of discussion and before unblinding of the study. All test scores considered as not valid are set to missing. If the comments indicate that all tests of the patient are invalid (e.g. patient is illiterate) the patient is excluded from CAROLINA®-cognition analysis. Furthermore impossible scores (e.g. VFT score after 60 s which is less than after 15 s) are also set to missing.

When baseline VFT and TMT scores are very low, deterioration over time cannot be reliably assessed due to floor effects. Therefore, patients with a baseline VFT score below 3 will not be considered for longitudinal analysis on the VFT and patients with a TMT ratio z-score of 2 or higher at baseline not for the longitudinal analyses on the TMT. In this case the composite score for attention and executive functioning is just based on the valid data.

### Cognitive outcomes

The primary outcome of CAROLINA®-cognition is the occurrence of accelerated cognitive decline at end of follow-up (a dichotomous outcome measure; presence or absence of accelerated cognitive decline).

Secondary cognitive outcomes are assessed as follows:

- The actual change in cognitive performance at end of follow-up (i.e. a continuous outcome measure; change in performance from baseline).
- The proportion of participants with accelerated cognitive decline after 160 weeks of follow-up.
- The actual change in cognitive performance after 160 weeks of follow-up (i.e. a continuous outcome measure; change in performance from baseline).

#### Primary outcome considerations

Conceptually, there are different ways to define accelerated cognitive decline. A fixed cut-off (e.g. occurrence of MMSE <24 at time point of assessment) or a minimal amount of decline (e.g. occurrence of >4 points of decline from baseline) can be used. However, a fixed cut-off does not take baseline performance into account and an absolute decline does not account for important individual factors influencing cognitive decline, such as education. We therefore choose to use a regression based index score (RBI score) of cognitive change over time. This RBI score adjusts for potential confounders as age, language, education, baseline performance, and regression to the mean on an individual participant basis [36]. In addition, the RBI also reduces the impact of learning effects: repeated neuropsychological assessment can cause practice effects, both material-specific effects and the fact that a person is no longer “test-naïve” after the first neuropsychological assessment. While the latter cannot be prevented, the former is countered by the use of RBI. Accelerated cognitive decline in the CAROLINA® cognition substudy is defined as a score at or below the 16th percentile (the equivalent of approximately one standard deviation below the mean) on the MMSE- or the A&E RBI z-score.

To convert MMSE and A&E z-scores into RBI scores, predicted follow-up scores (FUpredict) are calculated for each individual by means of an ANCOVA model. This model includes the following covariates: the individual’s baseline test performance, age, years of formal education, gender, race, and test–retest interval. Subsequently the RBI scores are calculated for each individual by comparing his/her actual observed cognitive (FUobserved) score to his/her predicted cognitive score (RBI-score = (FUobserved – FUpredict)/standard deviation (SD) of residuals). Hence, a negative RBI-score reflects a decline in cognitive function (relative to the other study participants) faster than expected (based on the adjusted covariates).

Clearly, dichotomizing the cognitive test results for the primary outcome measure does have implications for the analyses. It is also different from the approach of previous studies in the field [10–13]. Of note, our rationale for the dichotomy is that it has become apparent that cognitive decline in older individuals with T2DM is clearly not a unitary construct [2]. On average – at the group level - cognition declines only very slowly over time [10–13]. Yet, there is a subset of individuals with accelerated decline [2]. While ideally this accelerated cognitive decline would be defined in terms of incident dementia or mild cognitive impairment, this was not deemed to be feasible in the present multinational, multicenter study, because of variability in diagnostic approaches. We therefore choose the pragmatic approach as described above, which is likely to capture the patients with the worst cognitive outcome, although not in terms of a fixed diagnostic construct. Dichotomizing the cognitive test results based on the RBI could result in an underestimation of the standard error of the primary estimate of group difference in rate of cognitive decline. It also comes at the expense of information loss and power. Yet, it was decided to sacrifice some statistical power in order to enable the possibility of having a more powerful statement at the end of the trial. Moreover, the actual change in cognitive performance at end of follow-up (i.e. change in performance from baseline as a continuous measure) is an additional predefined outcome measure to confirm the results of the primary analysis.

#### Time windows

The time from baseline to end of follow-up cognitive assessment will vary between participants as patients were recruited over a period of two years. Furthermore, as visits may be rescheduled and each patient is followed up for a different time interval as per study design time windows were defined to assign each cognitive assessment to either baseline, week 160 or end of follow-up.

Baseline cognitive assessments were planned to be conducted at the day of randomisation, prior to intake of the first dose of study drug. The first follow-up assessment is scheduled after 160 weeks of follow-up (a time window up to 166 weeks is accepted) and the final cognitive assessment is scheduled within seven days after the last intake of study medication.

In practice the baseline test was conducted between Dec 2010 – Dec 2012 and the planned week 160 test was conducted between Dec 2013 – Jan 2016. The formal end of the trial will be determined in time, by reaching the predefined number of patients with primary endpoint events in the mother-trial, estimated to occur in Q2 2018. All patients that are still on treatment by then have their end of follow-up assessment at that time point. Patients that stop their treatment before the end of the trial will have their end of follow-up assessment at that moment. For all participants with a cognitive assessment after week 166, this assessment will be assigned to the second time interval (end of follow-up).

### Other study parameters

#### Demographics at baseline (full definitions listed in Additional file 2)

Demographic information is collected at baseline and include age, gender, years of formal education, race (Black/African American, White, Asian, American Indian/Alaska Native, Hawaiian/Pacific Islander), ethnicity (Latino/Hispanic, non-Latino/Hispanic), medication use, medical history, and alcohol use.

#### Diabetes-related variables

Blood samples are drawn at baseline and at the day of the first and second cognitive follow-up assessments and include, HbA1c, FBG, and C-peptide. Samples are always taken after an overnight fast (at least 10 h after the last meal) and all blood samples are analysed at a central laboratory using validated assays. Medical history is recorded in the case report form (CRF) and includes duration of diabetes and presence of diabetic complications (diabetic neuropathy, diabetic foot and proliferative retinopathy; full definitions listed in Additional file 2). Previous medication use, including SU or glinide is recorded. Episodes of hypoglycaemia, including severe hypoglycaemic episodes, are recorded prospectively.

#### Cardiovascular risk profile (full definitions listed in Additional file 2)

Cardiovascular risk factors are assessed at baseline and at the day of the first and second cognitive follow-up assessments. They include: smoking habits, systolic and diastolic blood pressure, body mass index (BMI), waist circumference, a lipid panel (total cholesterol, high density lipoprotein (HDL) cholesterol, low density lipoprotein (LDL) cholesterol, triglycerides), and assessment of renal function/albuminuria. Blood pressure is measured using either a standard mercury sphygmomanometer or an electronic device after five minutes of rest. Weight measurements are standardised and similar scales are used at each visit. Waist circumference is measured in the midpoint between the lowest rib and the iliac crest using a non-elastic tape, after the patient exhaled. Estimated glomerular filtration rate is calculated using the Modification of Diet in Renal Disease (MDRD) formula.

History of macrovascular disease includes: ischemic heart disease, cerebrovascular disease and peripheral arterial occlusive disease. Cardiovascular events are recorded prospectively.

### Statistical analysis

#### Sample size considerations

Accelerated decline is defined as an RBI score within the lowest 16% for the MMSE and/*or* the A&E RBI score. It is expected that an estimated of 20-22% will meet this criterion for the primary cognitive outcome measure of CAROLINA®. There were no formal power calculations performed for this substudy. However with 4500 participants, approximately 900–1000 participants will thus meet this primary cognitive outcome measure, which will allow, at a reasonable power, a detection of a hypothesised relative risk reduction with linagliptin for accelerated cognitive decline of approximately 20% (power 0.8; alpha 0.05, two-sided testing).

#### Primary analysis

The primary analysis will be performed in all patients randomised and treated with at least one dose of study drug, who have a baseline assessment and at least one follow-up cognitive assessment available (of which at least one of the two RBI scores can be calculated). In this modified intention to treat analysis the proportion of participants with accelerated cognitive decline will be compared between the two treatment groups at end of follow-up using a logistic regression analysis with factor for treatment. The odds ratio (OR) along with the 95% Wald confidence interval (CI) and the two-sided *p*-value for treatment comparison will be presented.

#### Predefined subgroup analyses

The primary outcome will be analysed in the following subgroups to explore the consistency of the treatment effect: gender (male, female), age (<70, ≥70 years), race (black, white), ethnicity (Latino/Hispanic, non-Latino/Hispanic), CES-D (score < 16, ≥ 16 and median split), cardiovascular risk groups (based on inclusion criterion groups A, B, C, D; see Table 1) and duration of diabetes (<=1 year, >1 to <=5 years, >5 to <=10 years, >10 years).

#### Handling of missing cognitive data

Missing baseline cognitive data will not be imputed. For missing data due to incomplete testing, the remaining test scores will be used to judge if accelerated cognitive decline is present. If one of the VFT subscores is missing, the remaining scores will be used to calculate the overall score. If either the TMT A or the TMT B is missing no TMT ratio will be calculated. If either the VFT overall z-score or the TMT ratio is missing the remaining score will be used to calculate the A&E score at baseline and follow-up.

If one follow-up assessment is completely missing it will be replaced by her/his last observed post-randomization measurement or linearly intrapolated in case of a missing assessment in between assessments.

If a cognitive test is not done or not completed, the investigator or research assistant should indicate whether this was due to the inability of the patient to understand the instructions. If this is the case at an follow-up visit and neither the MMSE nor the A&E RBI-score can be calculated due to missing values, the patient is classified as having accelerated cognitive decline.

#### Sensitivity analyses for the primary outcome

To test the robustness of the results, sensitivity analyses will be performed for the primary outcome (for the second FU assessment), as shown in Table 2.

#### Secondary analyses

To investigate potential early treatment effect, we will also look into the occurrence of accelerated cognitive decline at week 160, i.e., the first cognitive assessment post baseline.

In addition, to determine whether the definition we used for accelerated cognitive decline influenced the results, we will investigate the following alternative definitions for accelerated cognitive decline at week 160 and end of follow-up:

- having a score at or below the 16th percentile on the MMSE- or the A&E z-score (i.e. without using RBI scores).
- having a score at or below the 10th (instead of the 16th) percentile on the MMSE- or the A&E RBI-score
- having a follow-up MMSE score of <24 or a decline of >4 points in MMSE relative to baseline

To investigate the actual change in cognitive performance over time, the change in z-scores for all individual test scores (from baseline to first and second follow-up assessment) will be analysed. This will be done using a restricted maximum likelihood (REML) based mixed model repeated measures (MMRM) approach. The primary comparison will be the difference in adjusted LS means between the two treatment groups.

Finally, to investigate the effect of treatment on the occurrence of depression, the occurrence of a CES-D score of ≥16 will be analysed for the first and second follow-up assessments. This will be done using a logistic regression analyses, as for the primary outcome.

#### Exploratory analyses of risk factors for cognitive dysfunction

Additional analyses are planned to investigate the association between mood, diabetes-related factors, and cardiovascular factors and cognitive dysfunction. Cross-sectional baseline analyses will be conducted aimed at answering etiologic questions. Longitudinal analyses will be performed exploring both etiologic and prognostic questions in relation to cognitive decline.

Linear regression analyses will be used for the baseline analysis including the MMSE score and the A&E z-score as the cognitive outcome measures. These analyses will be adjusted for age, gender, years of formal education and race. If a significant association is found for a certain variable (e.g. HbA1c levels) other covariates may be added stepwise to the model to investigate this relation further. Non-linear associations will also be considered. We will perform subgroup analyses stratified by age (<70, ≥70 years) and gender (male, female). Similar approaches will be taken for etiologic longitudinal analyses, using a restricted maximum likelihood based mixed model repeated measures approach.

Since all of these secondary analyses are considered of exploratory nature, no correction for multiple testing will be made.

## Discussion

The CAROLINA® trial provides a unique opportunity to investigate the effect of treatment with linagliptin compared to the SU glimepiride on the occurrence of accelerated cognitive decline in patients with T2DM. The large sample size, the long follow-up period and the study population of middle aged and older (mean age 64.7 ± 9.4 years) individuals at elevated cardiovascular risk, offer an excellent cohort to study cognitive outcomes. With the primary outcome measure occurrence of accelerated cognitive decline the cognition study focuses on those individuals who suffer from cognitive problems; a novel and very clinically meaningful approach.

CAROLINA®-cognition, a substudy of the CAROLINA® trial, is the first large RCT that will yield important information regarding DPP-IV inhibitor versus SU treatment in the reduction of accelerated cognitive decline in patients with T2DM. A positive result in CAROLINA®-cognition could provide important leads towards a new prevention strategy for dementia in T2DM and as such have major clinical T2DM treatment ramifications.

## Additional files

Additional file 1: SPIRIT checklist. (DOCX 68 kb)

Additional file 2: Definitions of terms. (DOCX 19 kb)

## Abbreviations

A&E: Attention and Executive functioning
ANCOVA: Analysis of covariance
BMI: Body mass index
BP: Blood pressure
CABG: Coronary Artery Bypass Graft
CAROLINA: Cardiovascular Outcome Study of Linagliptin Versus Glimepiride in Patients With Type 2 diabetes mellitus
CES-D: Centre for Epidemiologic Studies Depression Scale
CI: Confidence interval
DPP-IV: Dipeptidyl peptidase-IV
GIP: Glucose-dependent insulinotropic polypeptide
GLP-1: Glucagon-like-peptide-1
HbA1c: Glycated hemoglobin
HDL: High Density Lipoprotein
LDL: Low Density Lipoprotein
MACE: Major Adverse Cardiovascular Event
MDRD: Modification of Diet in Renal Disease
MI: Myocardial Infarction
MMRM: Mixed model repeated measures
MMSE: Mini mental state examination
OR: Odds ratio
ORIGIN: Outcome Reduction With Initial Glargine Intervention
PCI: Percutaneous Coronary Intervention
RBI: Regression Based Index
RCT: Randomised controlled trial
REML: Restricted maximum likelihood
SMBG: Self-monitoring of blood glucose
SU: Sulfonylurea
T2DM: Type 2 Diabetes Mellitus
TMT: Trail Making Test
VFT: Verbal Fluency Test
