# Supplementary material for: Rationale and design of the CAROLINA® - cognition substudy: a randomised controlled trial on cognitive outcomes of linagliptin versus glimepiride in patients with type 2 diabetes mellitus
Source: BMC Neurol. 2018 Jan 15;18:7. doi: 10.1186/s12883-018-1014-7 (PMC5769408; doi:10.1186/s12883-018-1014-7)
Supplement: Supplementary file 2 — Definitions of terms. (DOCX 19 kb) [file 12883_2018_1014_MOESM2_ESM.docx]

| **Additional file 2 -** definitions of terms  **Baseline:** | |
| --- | --- |
| **Term** | **Definition** |
| Hypertension | Systolic blood pressure > 140 mmHg (or on at least one blood pressure lowering treatment) |
| Hypercholesterolemia | Current LDL cholesterol ≥ 135 mg/dL (3.5 mmol/l) (or specific current treatment for this lipid abnormality) |
| Smoking | Current daily cigarette smoking |
|  |  |
| Myocardial infarction | Myocardial infarction (> 6 weeks prior to informed consent) |
|  |  |
| Coronary artery disease | Documented coronary artery disease (≥ 50% luminal diameter narrowing of left main coronary artery or ≥50% in at least two major coronary arteries in angiogram) |
|  |  |
| Previous PCI  OR  Previous CABG  Ischemic heart disease  Macrovascular disease | Percutaneous Coronary Intervention (PCI) **>** 6 weeks prior informed consent  OR  Coronary Artery By-pass Grafting (CABG) > 4 years prior to informed consent or with recurrent angina following surgery  Includes myocardial infarction, coronary artery disease and previous PCI or CABG  Includes ischemic heart disease, cerebrovascular disease and peripheral occlusive arterial disease |
| Cerebrovascular disease | Ischemic or hemorrhagic stroke (> 3 months prior to informed consent) |
| Peripheral occlusive arterial disease | Includes: previous limb bypass surgery, stenting or percutaneous transluminal angioplasty; previous limb or foot amputation due to circulatory insufficiency, angiographic or ultrasound detected significant vessel stenosis (≥ 50%) of major limb arteries (common iliac, internal iliac, external iliac, femoral and/or popliteal artery), history of intermittent claudication with uni- or bilateral ankle: arm blood pressure ratio < 0.90) |
| Proliferative retinopathy | Retinal neovascularisation or previous retinal laser coagulation therapy |
| Renal impairment | Includes moderate and severe renal function impairment:   - Moderate renal function impairment: eGFR 30-59 mL/min/1.73m2 - Severe renal function impairment: eGFR <30 mL/min/1.73m2;   The modified diet of renal disease (MDRD) formula is used to estimate the glomerular filtration rate (eGFR) |
| Diabetic neuropathy  Microvascular complications | Based on patient’s medical history. Not further defined.  Includes proliferative retinopathy, renal impairment and diabetic neuropathy |
| Diabetic foot | Based on patients medical history. Not further defined |
| Depression | A score of 16 or more on the Center for Epidemiologic Studies Depression (CES-D) scale |
| **On treatment:** | |
| **Term** | **Definition** |
| Hypoglyceamic episodes | Includes e.g.:   - Documented hypoglycaemia with glucose concentration ≤ 70 mg/dl (≥ 3.0 mmol/l and ≤ 3.9 mmol/l) - Severe hypoglycaemic episode: event requiring the assistance of another person to actively administer carbohydrate, glucagon or other resuscitative actions |

| Cardiovascular events | Includes adjudicated events of:   - Non-fatal MI (excluding silent MI) - Hospitalisation for coronary revascularization procedures (CABG, PCI) - Non-fatal stroke - Hospitalisation for unstable angina pectoris - Transient ischemic attack - Hospitalisation for heart failure |
| --- | --- |
